# M^6^A modification in cardiovascular disease: With a focus on programmed cell death

**DOI:** 10.1016/j.gendis.2023.05.023

**Published:** 2023-07-14

**Authors:** Wen Li, Yao Liu, Ruiyan Xu, Yuan Zong, Lu He, Jun Hu, Guohua Li

**Affiliations:** a Institute of Cardiovascular Disease, Key Laboratory for Arteriosclerology of Hunan Province, Hunan International Scientific and Technological Cooperation Base of Arteriosclerotic Disease, Department of Pathophysiology, MOE Key Lab of Rare Pediatric Diseases, Hengyang Medical School, University of South China, Hengyang, Hunan 421001, China; b Department of Cardiovascular Medicine, The Second Affiliated Hospital, Hengyang Medical School, University of South China, Hengyang, Hunan 421001, China; c Department of Obstetrics and Gynecology, The First Affiliated Hospital, Hengyang Medical School, University of South China, Hengyang, Hunan 421001, China; d Department of Cardiovascular Surgery, The Second Affiliated Hospital, Hengyang Medical School, University of South China, Hengyang, Hunan 421001, China

**Keywords:** Apoptosis, Autophagy, Cardiovascular diseases, Ferroptosis, N6-methyladenosine, Programmed cell death, Pyroptosis

## Abstract

N^6^-methyladenosine (m^6^A) methylation is one of the most predominant internal RNA modifications in eukaryotes and has become a hot spot in the field of epigenetics in recent years. Cardiovascular diseases (CVDs) are a leading cause of death globally. Emerging evidence demonstrates that RNA modifications, such as the m^6^A modification, are associated with the development and progression of many diseases, including CVDs. An increasing body of studies has indicated that programmed cell death (PCD) plays a vital role in CVDs. However, the molecular mechanisms underlying m^6^A modification and PCD in CVDs remain poorly understood. Herein, elaborating on the highly complex connections between the m^6^A mechanisms and different PCD signaling pathways and clarifying the exact molecular mechanism of m^6^A modification mediating PCD have significant meaning in developing new strategies for the prevention and therapy of CVDs. There is great potential for clinical application.

## Introduction

Cardiovascular diseases (CVDs) encompass a variety of diseases, including coronary cardiac hypertrophy, heart failure, heart disease (CHD), and hypertension. Despite advances in the prevention, diagnosis, and early intervention of CVDs, it remains the primary cause of morbidity and mortality worldwide.^1^^,^^2^ Furthermore, CVDs are well-studied and highly correlated with biochemical, environmental, behavioral, geographic, and genetic factors through multiple rich, longitudinal, transgenerational, and deeply phenotypic cohort surveys.^3^, ^4^, ^5^ Therefore, understanding the molecular mechanism underlying CVDs occurrence and development has essential clinical significance for the early detection, treatment, and prognosis of CVDs.

Both genetic and epigenetic processes contribute to CVDs. With the increasing attention to epigenetics in recent years, emerging evidence has suggested the essential effect of epigenetic modifications in pathological CVDs.^6^ Epigenetics, including DNA methylation, RNA methylation, histone modifications, and noncoding RNA molecules, of which methylation is the only modification known to occur on the three central dogma molecules of DNA, RNA, and protein.^7^^,^^8^ As RNA-based CVD therapies have advanced significantly over the past decade,^9^ RNA methylation appears to be a prospective new addition to the field and has gained increasing public attention.^10^ It has been reported that RNA methylation consists of more than 170 different types of RNA modifications, mainly in the form of N6-methyladenosine (m^6^A), 5-methylcytosine, N7-methylguanosine, 5-hydroxymethylcytosine, N1-methyladenosine, 2′-O-dimethyladenosine, and 2′-O′ methylation.^11^ M^6^A is well depicted as the most abundant internal chemical modification of eukaryotic RNA transcripts.^12^ To date, there have been reports that m^6^A can be found in messenger RNA (mRNA), ribosomal RNA, transfer RNA (tRNA), small nucleolar RNA, long non-coding RNA, circular RNA, and microRNA.^13^ Several studies have identified aberrant m^6^A methylation in CVDs,^14^ such as atherosclerosis,^15^ cardiac hypertrophy,^16^^,^^17^ heart failure,^18^ myocardial ischemia-reperfusion injury,^19^ aneurysm,^20^ vascular calcification,^21^ diabetic cardiomyopathy (DCM),^22^ and pulmonary hypertension.^23^

Programmed cell death (PCD) is a significant pathophysiology signature of CVDs. It is also a major factor in maintaining normal cellular morphology, function, and the clearance of abnormal cells.^24^^,^^25^ Currently, known cell death modalities include, but are not limited to, apoptosis, autophagy, pyroptosis, ferroptosis, and necroptosis.^26^^,^^27^ It is important to understand how the extracellular microenvironment and intracellular gene regulation of PCD affect the behavior of cardiomyocytes and other cell types, such as endothelial cells and smooth muscle cells.

To provide the latest information for the clinical treatment of CVDs. This article reviews the mechanistic link between m^6^A and PCD in CVDs. Firstly, we comprehensively summarized the regulatory factors and biological functions of m^6^A, then systematically discussed the mechanisms of m^6^A-mediated various cell death modalities in different types of CVDs.

## M^6^A methylation regulators and functions

**Figure 1:**
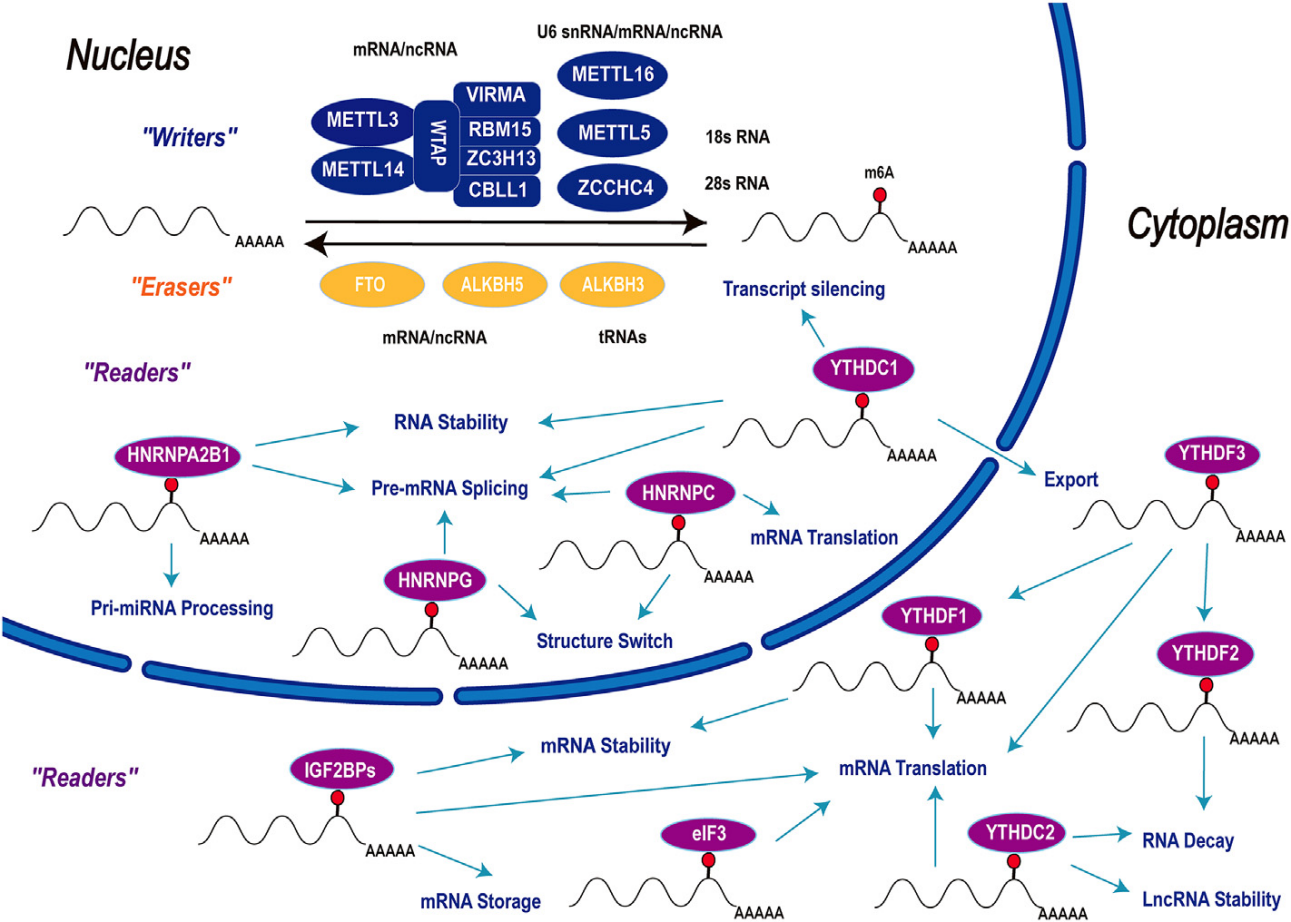
M^6^A RNA regulatory diagram. The m^6^A methylation modification process is dynamically reversible and requires the participation of writers, erasers, and readers. The primary role of writers is to methylate RNA sites, which mainly works by a writer complex consisting of the core subunits METTL3 and METTL14 and other adaptors proteins WTAP, VIRMA, RBM15/15B, ZC3H13, and CBLL1. Other known writers include METTL16, METTL5, and ZCCHC4. Erasers are responsible for demethylation, including FTO, ALKBH5, and ALKBH3. Readers selectively function to recognize by utilizing different mechanisms, consisting of YTHDF1–3, YTHDC1–2, IGF2BPs, eIF3, and HNRNPs, and their molecular biological functions including RNA splicing, translation, stability, decay, export, structure switch, and transcript silencing.

The abundance and effects of m^6^A on RNA are determined by the dynamic interplay between its methyltransferases (“writers”), binding proteins (“readers”), and demethylases (“erasers”).^28^ Writers, erasers, and readers are responsible for the occurrence, removal, and recognition of m^6^A RNA modification, respectively (Fig. 1).

### M^6^A methylation regulators

#### M^6^A writers: methyltransferases

M^6^A writers are a class of methyltransferases, consisting of methyltransferase-like 3 (METTL3), methyltransferase-like 5 (METTL5), methyltransferase-like 16 (METTL16), zinc finger CCHC-type containing 4 (ZCCHC4), and additional partner proteins such as methyltransferase-like 14 (METTL14), Wilms tumor 1-associating protein (WTAP), Vir-like m^6^A methyltransferase-associated (VIRMA; also known as KIAA1429), RNA-binding motif 15/15B (RBM15/15B), zinc finger CCCH-type containing 13 (ZC3H13), and Casitas B-lineage lymphoma-transforming sequence-like protein 1 (CBLL1; also known as Hakai).^29^, ^30^, ^31^, ^32^

METTL3 is the key protein of the m^6^A methyltransferase complex. It mainly functions as a catalytic subunit. As a synergistic partner for METTL3, METTL14 facilitates RNA binding by providing structural support for METTL3 when they combine into a core methyltransferase complex.^33^, ^34^, ^35^ WTAP is required to recruit optimal substrate and to localize METTL3/METTL14 complex.^36^^,^^37^ VIRMA regulates preferential methylation of m^6^A mRNA in 3′ untranslated regions (UTR) and near stop codon, while CBLL1 affects m^6^A modifications located in 5′UTRs and around the start codon.^32^^,^^38^ RBM15 and RBM15B bind to the methyltransferase complex and promote its recruitment to specific sites in RNA.^39^ ZC3H13 facilitates m^6^A methylation by anchoring WTAP, VIRMA, and CBLL1 in the nucleus.^40^ However, it is unclear why the m^6^A modification requires such a large complex, nor what each component does. Among all the m^6^A methyltransferases, METTL16, METTL5, and ZCCHC4 function independently of other methyltransferase proteins. METTL16 prefers binding a UAC (m^6^A) GAGAA sequence in U6 snRNA, inducing m^6^A deposition on nuclear RNA.^41^^,^^42^ METTL5 is responsible for 18S rRNA m^6^A modification and causes m^6^A on ribosomal RNA. ZCCHC4 is the 28S rRNA modification enzyme.^29^^,^^31^^,^^43^

#### M^6^A erasers: demethylases

M^6^A erasers are a class of demethylases acting via active demethylation by reversing and controlling the methylation-dependent processes, containing the fat mass and obesity-associated protein (FTO) and AlkB homolog 5 (ALKBH5) so far.^44^^,^^45^ FTO demethylates mRNA m^6^A and cap m^6^Am in the cytoplasm and demethylates mRNA m^6^A in the cell nucleus, which is essential for mRNA processing.^46^ ALKBH5 demethylates the m^6^A-containing ssRNA, similar to FTO, and plays a significant role in the export and metabolism of nuclear RNA.^44^ Mechanistically, FTO and ALKBH5 can mediate the oxidation of m^6^A to N^6^-hydroxymethyl adenosine (hm^6^A), which is further oxidized to N^6^-formyladenosine (f^6^A), and f^6^A is finally hydrolyzed to adenine, in a stepwise manner.^47^^,^^48^ As a kind of m^6^A demethylase of tRNA, ALKBH3 has only been ascertained in recent years, with the function of enhancing protein translation efficiency,^49^ serving as a potential direction for future research.

#### M^6^A readers: m^6^A-binding proteins

M^6^A readers can recruit diverse regulatory or functional machineries to the target m^6^A-containing mRNA, including YT521-B homology (YTH) domain family 1-3 (YTHDF1-3), YTH domain containing 1-2 (YTHDC1-2), insulin-like growth factor 2 mRNA-binding protein1-3 (IGF2BP1-3), eukaryotic initiation factor 3 (eIF3), and heterogeneous nuclear ribonucleoproteins (HNRNPs, including HNRNPA2B1, HNRNPC, and HNRNPG). YTHDF1/3 enhances RNA translation efficiency by promoting ribosome assembly and interacting with the initiation factor, YTHDF1 augments mRNA stability with m^6^A, and YTHDF2 induces the degradation of transcripts by directing its entry into bodies (P bodies) in the cytoplasm.^50^, ^51^, ^52^, ^53^, ^54^, ^55^ YTHDF2 recruits the CCR4-NOT deadenylase complex to increase the deadenylation activity and induce mRNA decay, and YTHDF2-mediated RNA processing is primarily responsible for gene expression (molecular function), cell death, and survival.^51^^,^^56^ YTHDF3 initiates circRNAs translation with eIF4G2.^57^ YTHDF3 also affects decay of methylated mRNAs associated with cooperation with YTHDF3 and YTHDF2.^54^ YTHDC1 can regulate mRNA splicing through the recruitment and combination of a certain splicing factor serine/arginine-rich splicing factor 3 (SRSF3) while blocking SRSF10 mRNA binding.^58^ YTHDC1 was also found to be involved in nuclear exporting with its YTH domain.^59^ Besides, YTHDC1 facilitates lncRNA X-inactive specific transcript (Xist)-mediated X chromosome silencing through m^6^A,^39^^,^^60^^,^^61^ and YTHDC1 maintained mRNA stability and reduced lncRNA stability.^62^^,^^63^ YTHDC2 facilitates both mRNA translation and decay, but promotes lncRNA stability, and also regulates spermatogenesis.^64^, ^65^, ^66^, ^67^ IGF2BPs affect gene expression output by promoting the stability, translation, and storage of the target mRNAs with m^6^A.^68^ Besides, IGF2BP2 is necessary for preserving the function of hematopoietic stem cells.^69^ EIF3 participates in translation initiation and termination, and ribosomal recycling. EIF3 can mediate selective internal initiation by directly binding to structured or chemically modified 5′UTRs in target mRNAs and plays a role in translation.^70^^,^^71^ HNRNPA2B1 reinforces mRNA stability and promotes mRNA nucleocytoplasmic trafficking, and it can directly bind to transcripts and trigger alternative splicing effects similar to the m^6^A writer METTL3, promoting primary miRNA processing.^72^, ^73^, ^74^, ^75^ HNRNPC interacts with coding and non-coding RNAs which have post-transcriptional m^6^A modifications (called “m^6^A-switch”), affecting the abundance as well as alternative splicing of target mRNAs. Recent studies have found that HNRNPC also plays a role in the translational regulation of mRNA target sites.^76^^,^^77^ HNRNPG also acts through “m^6^A-switch” and it works in the modulation of m^6^A-modified pre-mRNAs splicing with RNA polymerase II.^78^^,^^79^ Dynamic modifications of m^6^A are recognized by selective binding proteins, thereby affecting the translation status and lifespan of mRNAs. Thus, writers, erasers, and readers dynamically modulate m^6^A modification. As the most abundant mRNA modification, m^6^A could regulate multiple biological processes.

### Biological functions of m^6^A modification

The deposition of m^6^A RNA modification occurs co-transcriptionally and depends upon the transcribing RNA polymerase II (RNAPII) dynamics. Transcription efficiency affects the differential methylation profile in mRNA, which in turn affects translational efficiency.^80^ It is demonstrated that m^6^A on the RNA acts as a key regulatory module negatively associated with translational efficiency. With the rapid development of bioinformatic analyses and high-throughput sequencing technologies, m^6^A modification has been identified that mainly occurs at the consensus motif RR (m^6^A) CH (R = A or G; H = A, C, or U) in long internal exons, near stop codons, or in the 3′UTR.^81^^,^^82^ The variation in m^6^A levels is due to cis-regulation as well as extensively regulated by trans-factors.^83^^,^^84^ Not only does the m^6^A modification occur on mRNAs, but it is a widely recognized fact that m^6^A modification is also widespread in non-coding RNAs (ncRNAs).^85^^,^^86^ M^6^A modification is significant for the biogenesis of at least a subset of miRNAs, and/or for miRNA stability.^87^ M^6^A methylation promotes lncRNA mediated transcriptional repression,^88^^,^^89^ and the m^6^A level of lncRNA can be used as a new specific target for disease treatment and diagnosis.^90^ It is shown that m^6^A methylation regulates the biogenesis, stability, and translation of circular RNAs (circRNAs), controls circRNAs immunity, and impacts the biological functions of circRNAs.^57^^,^^91^^,^^92^ However, the most of current m^6^A studies focus on mRNA, and little is known about the functions and mechanisms of m^6^A-modified ncRNAs. In conclusion, m^6^A methylation plays an important role in regulating gene expression in the occurrence and development of various diseases.

## The role of m^6^A modification in PCD

**Figure 2:**
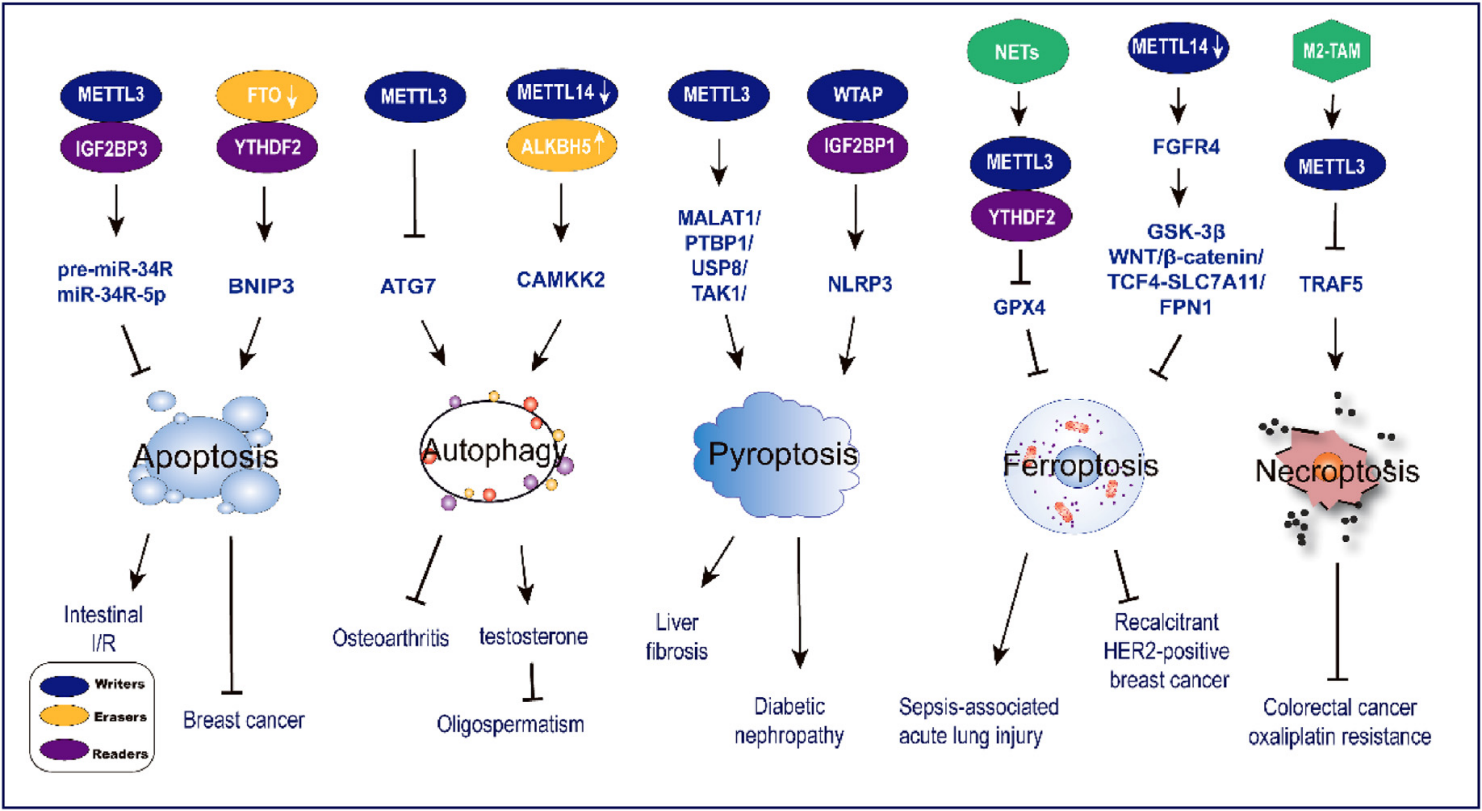
M^6^A and PCD. The dysregulation of m^6^A modification contributes to the pathogenesis of various diseases by affecting methyltransferases, demethytransferases, and binding protein activity. M^6^A modification inhibits intestinal ischemia-reperfusion (I/R) and breast cancer by regulating apoptosis and promotes osteoarthritis by suppressing autophagy. Down-regulated m^6^A modification accelerates autophagy to boost testosterone production and thus inhibits oligospermatism. Enhanced m^6^A modification accelerates pyroptosis to facilitate liver fibrosis and diabetic nephropathy, and aggravates sepsis-associated acute lung injury via ferroptosis. Down-regulated METTL14 suppresses ferroptosis to accelerate recalcitrant HER2-positive breast cancer. METTL3 inhibits necroptosis to promote colorectal cancer oxaliplatin resistance.

Since first demonstrated to be associated with apoptosis by regulating gene expression, plenty of evidence has been demonstrated that m^6^A modification interacts and affects with almost all types of PCD signaling pathways and cell survival (Fig. 2).

### M^6^A and apoptosis

Apoptosis is a process of spontaneous and orderly cell death characterized by the activation of cysteine proteases known as caspases, which have the endogenous mitochondrial pathway, the exogenous death receptor pathway, and the endoplasmic reticulum stress-mediated apoptosis pathway. Some researchers have shown that m^6^A affects apoptosis.^93^

A recent study reported that mesenchymal stem cells (MSCs) secreted exosomal miR-34a-5p via METTL3/IGF2BP3-mediated pre-miR-34A-m^6^A modification and ameliorated intestinal ischemia-reperfusion injury *in vitro* and *in vivo* experiments.^94^ Conversely, METTL14 was up-regulated in the ox-LDL-incubated human umbilical vein endothelial cells and facilitated P65 expression, thus playing a role in apoptosis and atherosclerosis.^95^ This implies that the fundamental role of m^6^A is disease- and phenotype-dependent. Furthermore, m^6^A demethylases play an indispensable part in the apoptosis pathway. As shown by Niu et al,^96^ they validated that silencing FTO can inhibit breast tumor growth. They dissected the molecular mechanism that silencing FTO increased BNIP3 expression by mediating m^6^A modification in the YTHDF2-independent manner, consequently strengthening breast cancer cell apoptosis. Different results in blocking demethylase activity were observed by Yu et al.^97^ This is the well-known phenomenon that faithful maintenance of genomic integrity plays an essential part in cell survival. During reactive oxygen species (ROS)-induced stress, ALKBH5 was thought to be the key regulator that protects cells from DNA damage and apoptosis. ROS strengthened the SUMOylation of ALKBH5 and enhanced the total level of m^6^A mRNA by activating the ERK/JNK pathway, and then improved ROS-induced DNA damage repair and suppressed apoptosis. The ERK/JNK/ALKBH5-PTMs/m^6^A axis plays a physiological part in maintaining genome integrity in mammalian cells in response to ROS.

Taken together, these results showed that m^6^A is positively associated with cell apoptosis and m^6^A-mediated apoptosis acts in tissue damage, which seems to have pathologic and therapeutic implications. Thus, there is an urgent need to learn about the molecular mechanisms which accelerate disease development by m^6^A-mediated apoptosis.

### M^6^A and autophagy

Autophagy is an important molecular pathway to maintain cellular and organismal homeostasis regulated by autophagy-related gene (ATG) proteins. Because autophagy is associated with many diseases, understanding how autophagy is regulated is becoming increasingly important. However, autophagy transcriptional regulatory mechanisms are currently still not well understood.^98^

A recent study has shown that m^6^A modification alters the transcriptional regulation of ATG proteins and may affect autophagy.^99^ FTO demethylase was observed to split UNC-51-like kinase 1(ULK1) from YTHDF2, increase ULK1 expression and enhance its levels, thus promoting autophagy. Since then, many articles have proved the roles of m^6^A modification in the autophagic process, such as in autophagosome formation and autophagy regulation.^100^ Mechanistic target of the rapamycin complex 1 (mTORC1) can restrain autophagy by phosphorylation of ATG13. Recent research has uncovered that m^6^A participates in the mTORC1 signaling cascade and that mTORC1 inhibits autophagy by activating the chaperonin-containing tailless complex polypeptide 1, which stabilizes the methyltransferase complex (METTL3/METTL14), resulting in elevated m^6^A levels of ATG gene mRNA and causing its degradation.^101^ Chen et al discovered that m^6^A modification is strongly connected with autophagy in osteoarthritis. METTL3-mediated m^6^A modification reduces ATG7 gene expression by reducing its RNA stability, resulting in impaired autophagy in osteoarthritis-related fibroblast-like synoviocytes.^102^ Song et al revealed that elevated METTL3 levels suppressed autophagic flux and promoted cell apoptosis, but overexpression of transcription factor EB (TFEB) or ALKBH5 reversed the effect of METTL3 on hypoxia/reoxygenation (H/R)-treated cardiomyocytes. The finding of an innovative regulatory mechanism for this lysosomal autophagy pathway provides a fresh approach to the treatment of ischemic diseases.^103^ Recent research has indicated that reduced METTL14 levels and increased ALKBH5 levels affect calcium/calmodulin-dependent protein kinase 2 (CAMKK2) mRNA stability and the translation of protein phosphatase magnesium-dependent 1A (PPM1A), and then activate the adenosine-5-monophosphate-activated protein kinase (AMPK) and the active regulator of autophagy ULK1 complex to initiate autophagy which stimulates testosterone production, thus avoiding oligospermia.^104^ Based on the above results, the link between m^6^A methylation and autophagy may provide novel therapeutic targets, which have important clinical implications.

### M^6^A and pyroptosis

Pyroptosis was defined as gasdermin-mediated and caspase 1-dependent programmed death. It was characterized by pathways that activate NOD-like receptors, specifically the NOD-like receptor thermal protein domain associated protein 3 (NLRP3) inflammasome as well as its downstream effector inflammatory factors interleukin (IL)-1β and IL-18. Normally, caspase-1 cleaves gasdermin D through an inflammatory pathway, leading to the pyroptosis of immune cells. Studies have revealed that m^6^A modification affects the regulatory pathway responsible for pyroptosis.^105^

Previous research has illustrated that m^6^A modification is involved in pyroptosis during hypothermia protection of neurons from ischemia/reperfusion. Mechanistically, hypothermia up-regulated phosphatase and tensin homolog (PTEN) mRNA levels through m^6^A modification, inhibited PI3K/Akt/GSK-3β signaling pathway, and down-regulated NLRP3 expression, thereby inhibiting pyroptosis.^106^ Shu et al reported that METTL3 activates MALAT1/PTBP1/USP8/TAK1 axis, facilitates hepatic macrophage pyroptosis and M1 hepatic macrophage polarization, and aggravates liver fibrosis.^107^ This will help to investigate the importance of METTL3 in other cell types and throughout the liver. It was also shown that METTL14-mediated m^6^A modification is connected with the pyroptosis of DCM. Down-regulation of METTL14 reduces the degradation of lncRNA TINCR, which positively regulates NLRP3 by enhancing its mRNA stability, thereby promoting pyroptosis in DCM rats.^108^ This discovery provides a better grasp of the regulatory network in myocardial injury, and it is expected to bring new opportunities for the therapeutic options of DCM by manipulating METTL14. According to Chen et al, WTAP directly modulates the m^6^A methylation of NLRP3 mRNA and maintains its RNA stability through IGF2BP1. This leads to the activation of NLRP3 inflammasome, which further triggers pyroptosis and inflammation of diabetic nephropathy.^109^ Depending on various conditions, pyroptosis modulation by these m^6^A modifications might represent a bi-directional pathway to protect cells against disease.

Despite the growing recognition of the biological significance of m^6^A modification, the overall impact of m^6^A-regulated factors on the transcription and translation of more pyroptosis-related genes remains poorly understood. These discovery inadequacies highlight the need for further research in this area.

### M^6^A and ferroptosis

Ferroptosis is a PCD characterized by iron accumulation and lipid peroxidation, independent of the caspase pathway.^110^ So far, ferroptosis has been confirmed to play an essential role in various diseases and has become the research focus.^111^

Ferroptosis can be induced by suppressing glutathione peroxidase 4 (GPX4). Recent research identified the relationship between ferroptosis and m^6^A modification. The release of neutrophil extracellular traps is one of the main strategies used by polymorphonuclear neutrophils to attack microbes. They activate TLR9/MyD88/NF-kβ pathway, up-regulate METTL3 expression, and promote m^6^A modification of GPX4, resulting in reduced GPX4 expression and triggering alveolar epithelial cell ferroptosis in the pathogenesis of sepsis-induced acute lung injury.^112^ The examination provides a potential novel therapeutic strategy for sepsis-associated lung injury by blocking METTL3, targeting neutrophils and neutrophil extracellular traps with the corresponding inhibitors. Furthermore, a novel assay analyzed the association of recalcitrant human epidermal growth factor receptor-2 (HER2)-positive breast cancer with ferroptosis. The down-regulation of METTL14 leads to reduced fibroblast growth factor receptor 4 (FGFR4) mRNA decay through m^6^A RNA hypomethylation, thus initiating the β-catenin/TCF4-SLC7A11/FPN1 pathway to develop anti-HER2 resistance in breast cancer by attenuating ferroptosis.^113^ This finding offers a promising opportunity for reverse anti-HER2 resistance by inhibiting the expression of FGFR4 and promoting ferroptosis in recalcitrant HER2-positive breast cancer. Another academic research has elucidated the regulatory mechanism of METTL14 on ferroptosis in myocardial injury. The up-regulation of METTL14 catalyzed the m^6^A modification of the long non-coding RNA KCNQ1OT1, which increased its stability by binding to IGF2BP1, substantially inhibiting miR-7-5p activity. Moreover, the lack of miR-7-5p expression will increase the transferrin receptor level, promoting iron uptake and production of reactive oxygen species, ultimately initiating DOX-induced ferroptosis and cardiotoxicity.^114^

The current results provide information on the value of the regulation of m^6^A targeting ferroptosis; however, less is known regarding its interaction with human diseases. Therefore, further research is certainly warranted.

### M^6^A and necroptosis

Necroptosis is also a caspase-independent form of PCD performed by the receptor-interacting protein kinase 1 (RIPK1)-RIPK3-mixed lineage kinase domain-like protein (MLKL) signaling cascade and is triggered by downstream of death domain receptors (*e.g.*, tumor necrosis factor receptors TNFR and Fas) and Toll-like receptors 3/4 (TLR3/4).^115^

A previous study about oxaliplatin for advanced colorectal cancer detected the link between m^6^A and necroptosis. M2-tumor-associated macrophages promote METTL3 expression and increase the m^6^A RNA content of TNF receptor-associated factor 5 (TRAF5), affecting the tumor microenvironment, promoting necroptosis, and leading to acquired oxaliplatin tolerance.^116^ The bioinformatics analysis for necroptosis-related lncRNAs (nrlncRNAs) to give predictions for the prognosis of neck squamous cell carcinoma, suggested the correlation of nrlncRNAs signature with m^6^A.^117^ However, the bioinformatics analysis still needs prospective research with experimental investigations to confirm the results. Furthermore, m^6^A modification in necroptosis has rarely been studied. Therefore, the detailed regulatory mechanism remains unclear.

As previously described, dysregulated expression of m^6^A regulators is involved in disorders of various cell death processes. M^6^A modification plays a double-edged role in multiple condition-related cell death processes, which may depend on the specific sequence binding preference for m^6^A regulators as well as the transcription factors.

## M^6^A-regulated PCD in CVDs

**Figure 3:**
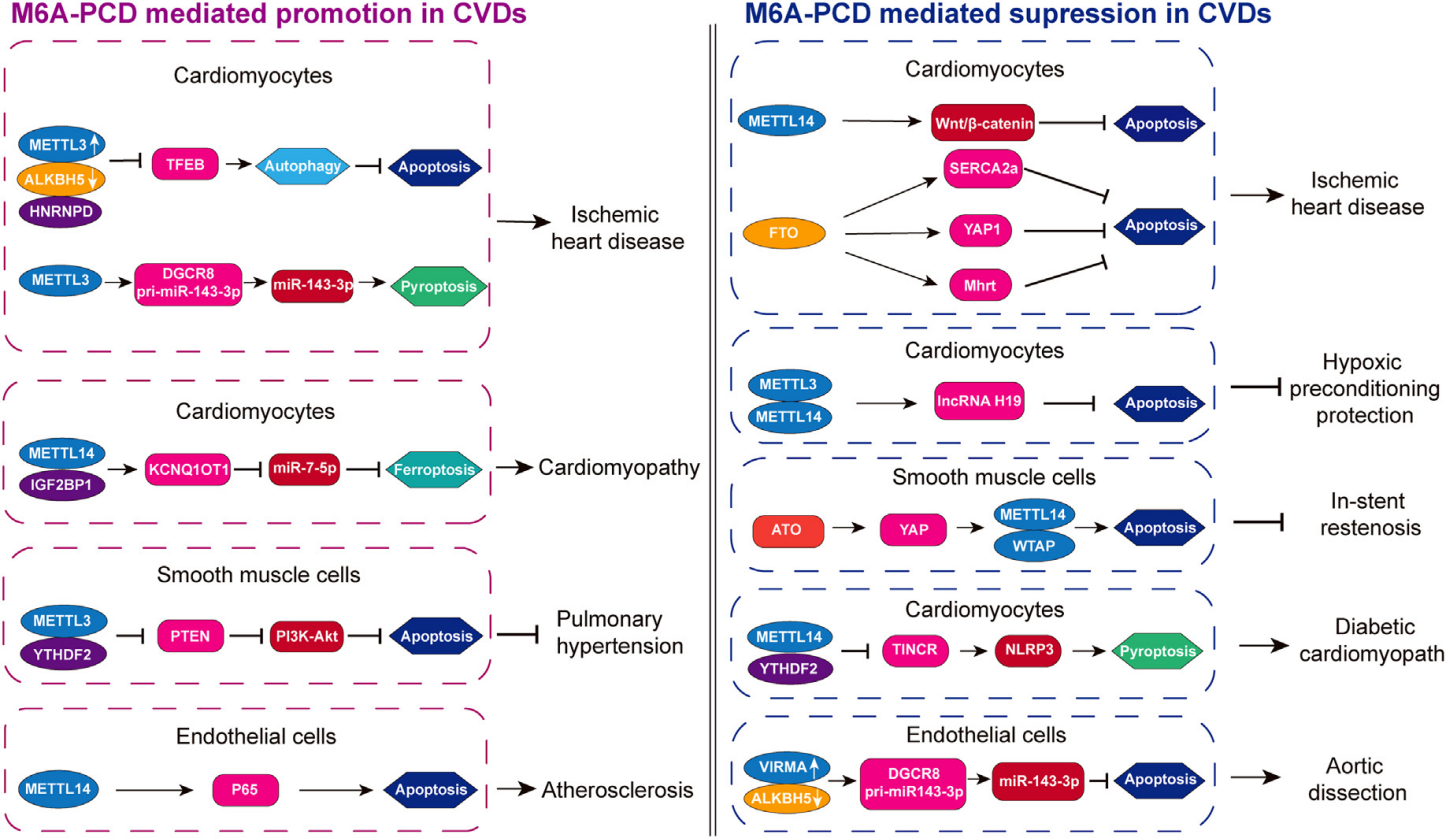
M^6^A and PCD in CVDs. M^6^A-mediated PCD in cardiomyocytes, smooth muscle cells, and endothelial cells promotes or inhibits the development of CVDs.

As described above, m^6^A modification is tightly related to multiple diseases through PCD pathways. With further research, the m^6^A-PCD axis would act as a critical part in the occurrence and progression of CVDs by exerting either protective or detrimental effects (Fig. 3).

### M^6^A-PCD axis promoting CVDs

The m^6^A methylation process mainly affects cardiomyocytes, vascular smooth muscle cells (VSMCs), vascular endothelial cells, and macrophages, and its methylation in these cells conduces to the occurrence and development of CVDs.

As previously described, METTL3 was up-regulated and ALKBH5 was down-regulated in ischemic heart disease, which reduces TFEB mRNA expression via catalyzing m^6^A methylation and accelerating the combination of HNRNPD and TFEB pre-mRNA, producing autophagy inhibition resulting in H/R induced cardiomyocyte apoptosis. This suggests that METTL3 behaves as a negative regulator of autophagy in ischemic heart diseases, while ALKBH5 functions oppositely.^103^ In addition, METTL3 enhanced the binding of DiGeorge syndrome critical region 8 (DGCR8) and pri-miR143-3p through m^6^A modification, causing the increase of mature miR-143-3p to repress protein kinase C epsilon transcription, further exacerbating cardiomyocyte pyroptosis and myocardial ischemia-reperfusion injury.^118^ Moreover, a contemporary study investigating hypoxic pulmonary hypertension argued that METTL3 expression is abnormally up-regulated through m^6^A modification in a YTHDF2-dependent manner, accelerating the degradation of PTEN mRNA and shortening its half-life. This leads to mediating the migration and proliferation of hypoxic pulmonary artery smooth muscle cells (PASMCs) and reducing apoptosis by activating PI3K/Akt signaling pathway.^119^ Based on the great number of findings, inhibition of METTL3 should be taken into account in the therapy of CVDs. Furthermore, METTL14 has been proven to control ferroptosis-associated cardiomyopathy.^114^ Moreover, METTL14 expression and m^6^A levels were significantly up-regulated by ox-LDL and other factors, affecting the stability of p65 and its expression, and vascular endothelial cells underwent abnormal apoptosis, leading to vascular dysfunction and promoting atherogenesis. However, the underlying molecular mechanisms are still not fully understood.^95^ This finding will contribute to further research on CVDs and develop effective treatment strategies for atherosclerosis.

### M^6^A-PCD axis inhibiting CVDs

The m^6^A methylation process also inhibits the progression of CVDs by affecting different kinds of PCD. A recent examination revealed that METTL14 promotes Wnt1 translation by enhancing m^6^A modification of its transcript, activates the Wnt/β-catenin pathway, and thus attenuates cardiomyocyte apoptosis and cardiac dysfunction induced by ischemia-reperfusion.^120^ Su et al demonstrated that METTL3 and METTL14 augment the enrichment of IncRNA H19 through m^6^A modification, consequently exerting an anti-apoptotic effect in the hypoxic preconditioning treatment of cardiac myocytes.^121^ How to inhibit VSMC proliferation without dysregulation of endothelial cells is an important challenge in overcoming in-stent restenosis. Arsenic trioxide (ATO) was found to induce VSMCs apoptosis but not affect endothelial cells. In terms of mechanism, ATO increases lysophosphatidic acid in VSMC through cellular metabolism, whereas lysophosphatidic acid is stable in endothelial cells. Lysophosphatidic acid activates yes-associated protein (YAP), induces METTL14 and WTAP expression, and up-regulates m^6^A methylation levels of apoptosis-related genes, thereby promoting VSMCs apoptosis,^122^ suggesting the enormous potential of ATO-eluting stents for the treatment of in-stent restenosis. Taken together, m^6^A writers, especially METTL14, were attested to exert cardioprotective effects by inhibiting cardiomyocyte apoptosis as well as vascular protective effects by promoting apoptosis of hyperproliferative VSMCs. Furthermore, METTL14 down-regulates lncRNA TINCR by elevating its m^6^A level in a YTHDF2-dependent manner and destabilizes NLRP3 mRNA through the TINCR-NLRP3 pathway, further inhibiting cardiomyocyte pyroptosis and DCM progression.^108^ Interestingly, contrary to previous myocardial ischemia-reperfusion research,^118^ VIRMA (also known as KIAA1429) also participates in the binding of DGCR8 to pri-miR143-3p, in which the target gene is DEAD-box helicase 6 (DDX6). However, this binding suppresses rather than promotes human aortic endothelial cell (HAEC) apoptosis and aortic dissection progression. Moreover, gain- and loss-of-function research exhibited that ALKBH5 has the opposite effect.^123^

Plenty of studies have proven the potential role of FTO in various CVDs. Recent studies have indicated that FTO-mediated demethylation can down-regulate the m^6^A level on SERCA2a mRNA, and enhance the SERCA2a mRNA stability and expression, thereby increasing the viability of cardiomyocytes and protecting cardiomyocytes from H/R-induced apoptosis.^124^ Additionally, FTO could uninstall m^6^A modification of YAP1 mRNA which acted as the target of FTO, mitigating the apoptosis and inflammatory reaction in cardiomyocytes from H/R injury.^125^ Moreover, FTO overexpression also inhibits myocardial cell apoptosis by regulating Mhrt m^6^A modification.^126^ In a word, FTO protects cardiomyocytes from H/R-induced apoptosis. The reduction in m^6^A levels due to FTO overexpression is beneficial to the heart and may improve cardiac dysfunction in ischemic cardiomyopathy, suggesting a potential application of FTO.

According to these findings, understanding the role of the dynamic regulation of m^6^A modification in modulating gene expression in cell death will not only help decipher the epigenome of CVDs associated with m^6^A but also offer new ideas for the diagnosis and therapy of disease. There is an urgent need to study PCD signaling pathways with m^6^A in various CVDs in the future.

## Clinical application of m^6^A-PCD axis in CVDs

A great deal of research has suggested that m^6^A-related genes, m^6^A modification regulators, and the m^6^A-medicated PCD axis positively or negatively adjusted the progression of CVDs. Due to the RNA containing plentiful m^6^A sites in the PCD pathway, targeting the interaction offers more promising therapeutic benefits than targeting individual m^6^A (Table 1).

**Table 1:** The clinical application of M^6^A and PCD in CVDs.

M^6^A regulators	Types of PCD	Molecular mechanisms	Cell type	The corresponding CVDs	Clinical applications	Treatment measures	Reference
METTL3	Apoptosis	Vitamin D3 rectified paradoxical m^6^A modification of MCU mRNA	Endothelial cells	Atherosclerosis	Therapy	METTL3 down-regulation	133
YTHDF3							
METTL3	Apoptosis	Diminished global m^6^A content and alleviated cardiac lipid accumulation	Cardiomyocyte	Obesity cardiomyopathy	Therapy	METTL3 down-regulation	135
FTO						FTO up-regulation	
METTL3	Pyroptosis	IRF-1 elevated the m^6^A level of circ_0029589	Macrophage	Acute coronary syndrome	Therapy	METTL3 down-regulation	127
YTHDF2							
METTL3	Ferroptosis	Reduced protein levels of SLC7A11 and FSP1	Smooth muscle cells	Aortic dissection	Therapy	METTL3 down-regulation	129
METTL14	Ferroptosis	Increased KCNQ1OT1 stability and inhibited miR-7-5p activity	Cardiomyocyte	Cardiomyopathy	Therapy	METTL14 down-regulation	114
IGF2BP1							
FTO	Apoptosis	Reduced the binding of miR-636 to DKK2 and promoted the expression of DKK2	Cardiac fibroblast	Myocardial fibrosis	Therapy	FTO up-regulation	130
FTO	Apoptosis	Exercise training mediated higher total m^6^A levels and down-regulated FTO protein levels	Cardiomyocyte	Heart failure with preserved ejection fraction	Therapy	FTO down-regulation	134
ALKBH5	Apoptosis	Up-regulated the expression of CDR1as and activated the Hippo signaling pathway	Cardiomyocyte	Diabetic cardiomyopathy	Therapy	ALKBH5 down-regulation	131
YTHDF2							
ALKBH5	Apoptosis	Promoted Bcl2 expression	Endothelial cells	Atherosclerosis	Therapy	ALKBH5 up-regulation	132

Recent research has revealed that METTL3 up-regulates m^6^A levels of circ_0029589 with the reader protein YTHDF2, reduces circ_0029589 expression, and mediates IFN regulatory factor 1 (IRF-1) pro-macrophage pyroptosis and inflammation in acute coronary syndrome.^127^^,^^128^ This work extends previous research and implicates down-regulating METTL3 or adjusting IRF-1 as a targeted therapy for the prevention and treatment of acute coronary syndrome. A recent examination revealed that METTL3 is up-regulated to reduce the protein levels of solute carrier family 7 member 11 (SLC7A11) and ferroptosis-suppressor-protein 1 (FSP1) by promoting their mRNA degradation, leading to facilitating ferroptosis of human aortic smooth muscle cells (HASMCs) and aortic dissection progression. The elucidation of this key mechanism provides a new strategy to target ferroptosis to treat aortic dissection.^129^ The suppression of lncRNA KCNQ1OT1 mitigates cardiomyopathy by attenuating the ferroptosis of cardiomyocytes. This may be achieved by lowering the lncRNA KCNQ1OT1 m^6^A methylation level.^114^ In a study of myocardial fibrosis, Li et al found that FTO down-regulated Dickkopf WNT signaling pathway inhibitor 2 (DKK2) m^6^A methylation, reduced the association of miR-636 to DKK2, promoted the DKK2 expression, and inhibited cardiac fibroblasts and myocardial apoptosis. These pathways may be significant pathways closely related to myocardial fibrosis and can serve as important targets for diagnosing and treating myocardial fibrosis-related diseases.^130^ Shao et al found that ALKBH5 post-transcriptionally activated forkhead box O3 (FOXO3) by demethylation of m^6^A via YTHDF2, enhanced cerebellar degeneration-related protein 1 antisense (CDR1as) expression, and then activated Hippo signaling pathway conducing to cardiomyocyte apoptosis in DCM mice.^131^ Silencing the ALKBH5-FOXO3 m^6^A-FOXO3 mRNA-CDR1as/Hippo signaling pathway may be a promising new treatment idea for DCM. ALKBH5 promotes Bcl2 expression, thereby attenuating TNF-α-induced human umbilical vein endothelial cell injury. It helps us understand better and offers potential treatment avenues for atherosclerosis.^132^

It has been demonstrated that pharmacotherapy, exercise, and diet therapy are involved in m^6^A-PCD pathways. During the development of human cytomegalovirus-infected atherosclerosis, vitamin D3 can negatively regulate METTL3, inhibit human cytomegalovirus-induced increase in m^6^A methylation of mitochondrial calcium uniporter (MCU) in a YTHDF3-dependent manner, thereby ameliorating cell apoptosis and exerting its endothelial protective function.^133^ The latest relevant research demonstrated that exercising training could mediate higher m^6^A levels by down-regulating FTO and reducing myocyte apoptosis, thereby ameliorating myocardial phenotypes in heart failure with preserved ejection fraction. This heralds that FTO acts as a potential therapeutic target for the regulation of cardiomyocyte function.^134^ Furthermore, it has revealed that intermittent fasting, a nutritional approach, diminishes global m^6^A content by down-regulating METTL3 expression and up-regulating FTO, alleviates cardiac lipid accumulation and apoptosis against high-fat diet (HFD)-induced obesity cardiomyopathy.^135^ Previous studies have demonstrated intermittent fasting could activate TFEB to rescue advanced R120G αB-crystallin mutant-induced cardiomyopathy, but whether m^6^A-mediated autophagy participates in the regulation of metabolism remains unclear.^136^ Overall, this work provided previously unrecognized insights into the effects of intermittent fasting on m^6^A RNA methylation and may contribute to the development of non-pharmacological interventions for obese cardiomyopathy patients.

According to the current study, down-regulation of METTL3 or up-regulation of FTO can inhibit different types of cardiovascular-related cell PCD and thus inhibit the progression of CVDs. The development of new drugs targeting METTL3 and FTO may be more helpful to provide new directions for clinical treatment. With the continuous deepening of research on CVDs, more m^6^A modification regulators closely related to CVDs will be revealed, which may also become the targets for the prevention and treatment of CVDs. In general, m^6^A modification can affect different types of CVDs by regulating PCD such as apoptosis, autophagy, pyroptosis, and ferroptosis. However, there are no reports of m^6^A being associated with necroptosis in CVDs. By exploring its mechanism of action, it is expected to identify m^6^A regulators and upstream/downstream signaling molecules as targets for treating CVDs.

## Conclusion

With the development of society, understanding the pathogenesis of CVDs is advancing with the times. A number of studies have demonstrated that PCD plays a vital part in the pathogenesis of CVDs. Berulava et al found that approximately one-quarter of the transcripts in mouse and human hearts exhibit m^6^A RNA methylation, and high expression of m^6^A RNA methylation may contribute to the development of certain CVDs. In contrast, the low expression of m^6^A RNA methylation may lead to the progression of other CVDs.^18^ The functional effects of m^6^A on mRNA and carRNAs are diverse. Writers, erasers, and readers have their characteristics and each cannot replace the other, which leads to an extremely complex regulatory process. Since the dynamic process of methylation-demethylation is regulated in many aspects, the regulatory mechanisms of m^6^A can be improved by comprehensive assessment and discussion of the relevant phenotypes for specific biological processes and diseases. Undoubtedly, the correlation of m^6^A and PCD pathways will afford critical new insights into managing related diseases.

However, the current status of study on m^6^A and PCD is mainly in the field of oncology, and there are few reports related to CVDs. Among them, m^6^A exhibited conflicting roles, METTL3 promotes cardiomyocyte apoptosis while METTL14 inhibits cardiomyocyte apoptosis to affect the progression of ischemic cardiomyopathy,^103^^,^^120^ which would be due to different models of disease or differences in m^6^A mRNA status and function or different physiological and pathological environments. Different cellular functions regulated and controlled by target genes, different types of regulatory proteins that respond to m^6^A modifications, and different mRNA regions of m^6^A distribution may be plausible explanations for these contradictory phenomena. There are some limitations in the current studies, which should stimulate further research. One shortcoming is clinical samples are often in shortage. Another one is that researchers mainly investigated one or two cell types in CVDs. Besides, the differences in the regulatory effects of various m^6^A regulators on CVDs still need to be explored. In conclusion, m^6^A-mediated PCD of mRNA and ncRNA regulates the initiation and progression of CVDs, and exploring the correlation will be conducive to providing direction for developing better prevention, early diagnosis, and accurate personalized treatment for CVDs.

In addition, great progress has been made in the research of oncology drugs, but most of them have cardiovascular side effects. Timely provision of appropriate pharmacological prevention and intervention is an effective way to lessen the occurrence of cardiovascular toxicity and death caused by tumor treatment. With further research, nucleic acid methylation plays a vital part in the development of tumors and CVDs.^137^ Exploring their common methylation sites may lead to multiple optional targets for tumor therapy and the prevention and treatment of cardiovascular toxicity.

## Author contributions

Wen Li wrote and revised the paper; Yao Liu and Ruiyan Xu consulted literature and supplemented the article; Yuan Zong and Lu He drew diagrams and wrote captions; Jun Hu participated in writing the manuscript; Guohua Li designed, revised, and supervised the paper; all authors analyzed the literature and finished proofreading the papers.

## Conflict of interests

All authors declare no conflict of interests.

## Funding

This work was supported by the 10.13039/501100004735Hunan Provincial Natural Science Foundation of China (No. 2022JJ30502, 2022JJ30527), the Scientific Research Project of the Hunan Provincial Department of Education (China) (No. 20B493), and the Health Research Project of the Hunan Provincial Health Commission (China) (No. C202304027603).
